# Neutral polysaccharide from *Gastrodia elata* alleviates cerebral ischemia–reperfusion injury by inhibiting ferroptosis‐mediated neuroinflammation via the NRF2/HO‐1 signaling pathway

**DOI:** 10.1111/cns.14456

**Published:** 2023-09-26

**Authors:** Yonggang Zhang, Peng Ye, Hua Zhu, Lijuan Gu, Yuntao Li, Shi Feng, Zhi Zeng, Qianxue Chen, Benhong Zhou, Xiaoxing Xiong

**Affiliations:** ^1^ Department of Neurosurgery Renmin Hospital of Wuhan University Wuhan China; ^2^ Department of Pharmacy Renmin Hospital of Wuhan University Wuhan China; ^3^ Central Laboratory Renmin Hospital of Wuhan University Wuhan China; ^4^ Department of Neurosurgery, The Affiliated Huzhou Hospital Zhejiang University School of Medicine (Huzhou Central Hospital) Huzhou China; ^5^ Department of Pathology Renmin Hospital of Wuhan University Wuhan China

**Keywords:** ferroptosis, ischemic stroke, neuroinflammation, neutral polysaccharide from *Gastrodia elata*, NRF2/HO‐1 signaling pathway

## Abstract

**Aims:**

The crosstalk between ferroptosis and neuroinflammation considerably impacts the pathogenesis of cerebral ischemia–reperfusion injury (CIRI). Neutral polysaccharide from *Gastrodia elata* (NPGE) has shown significant effects against oxidative stress and inflammation. This study investigated the potential effects of NPGE on CIRI neuropathology.

**Methods:**

The effects of NPGE were studied in a mouse model of ischemic stroke (IS) and in oxygen–glucose deprivation/reperfusion (OGD/R)‐induced HT22 cells.

**Results:**

NPGE treatment decreased neurological deficits, reduced infarct volume, and alleviated cerebral edema in IS mice, and promoted the survival of OGD/R‐induced HT22 cells. Mechanistically, NPGE treatment alleviated neuronal ferroptosis by upregulating GPX4 levels, lowering reactive oxygen species (ROS), malondialdehyde (MDA), and Fe^2+^ excessive hoarding, and meliorating GSH levels and SOD activity. Additionally, it inhibited neuroinflammation by down‐regulating the level of IL‐1β, IL‐6, TNF‐α, NLRP3, and HMGB1. Meanwhile, NPGE treatment alleviated ferroptosis and inflammation in erastin‐stimulated HT22 cells. Furthermore, NPGE up‐regulated the expression of NRF2 and HO‐1 and promoted the translocation of NRF2 into the nucleus. Using the NRF2 inhibitor brusatol, we verified that NRF2/HO‐1 signaling mediated the anti‐ferroptotic and anti‐inflammatory properties of NPGE.

**Conclusion:**

Collectively, our results demonstrate the protective effects of NPGE and highlight its therapeutic potential as a drug component for CIRI treatment.

## Graphical abstract

Neutral polysaccharide from *Gastrodia elata* alleviates neuronal ferroptosis and ferroptosis‐induced neuroinflammation after I/R injury.

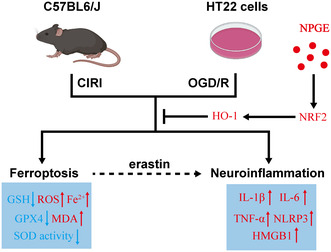

## 1 INTRODUCTION

Ischemic stroke (IS) results from arterial narrowing or occlusion, mainly in branches of the internal carotid artery. Although not the most lethal condition worldwide, IS remains a considerable challenge that needs to be addressed urgently because of the high disability rate in surviving patients.^1^ Early revascularization remains the only effective treatment, but it will result in cerebral ischemia–reperfusion (I/R) injury (CIRI), which has a complex pathological mechanism.^2^ In recent years, with the gradual deepening of our understanding of CIRI, targeting ferroptosis and neuroinflammation has become a promising therapeutic intervention.^3^, ^4^

Ferroptosis, discovered in recent years, refers to a programmed and gene‐regulated cell death process.^5^ Ferroptosis is activated after CIRI and the core mechanisms include lipid hyperoxidation and dysregulation of the antioxidant system.^5^ After I/R injury, the body produces large amounts of ROS, which promotes lipid peroxidation through the Fenton reaction; this process leads to the accumulation of MDA, which is one of the critical factors causing cell death.^6^, ^7^ Inhibition of lipid peroxidation is an important mechanism to suppress ferroptosis. Glutathione (GSH) is a crucial antioxidant regulator of ferroptosis. Its synthesis depends on the XC^−^ system, an essential subunit of which is solute carrier family seven member 11 (SLC7A11).^7^ Furthermore, the function of GSH is dependent on the core protein glutathione peroxidase 4 (GPX4), which is the only antioxidant enzyme that can convert GSH to GSSC and whose activation can effectively reverse ferroptosis.^6^, ^7^ Studies have shown that targeting ferroptosis can significantly reduce CIRI and improve the outcome of IS.^5^

Active ingredients derived from natural plants exhibit excellent anti‐inflammatory and antioxidant properties and are promising agents for the management of IS.^8^ *Gastrodia elata*, a plant that has been used in traditional Chinese medicine, has multiple biological activities, including antioxidant, and anti‐neuroinflammatory.^9^, ^10^ *G. elata* protects neuronal cells against CIRI, a function associated with its antioxidant and anti‐inflammatory activities.^11^ Neutral polysaccharide from *G. elata* (NPGE) is a component isolated from the plant; it is ~12 kDa and has antioxidant, immunomodulatory, and neuroprotective effects.^12^, ^13^, ^14^, ^15^ We previously found that NPGE treatment protects PC12 cells against corticosterone‐induced damage by inhibiting the endoplasmic reticulum stress‐related pathway,^16^ and reduces the expression of IL‐6, IL‐8, TNF‐α, and IL‐1β in a neuropathic pain model.^17^ Whether NPGE is neuroprotective in the acute phase of CIRI remains unknown.

In this study, we investigated for the first time whether NPGE treatment exerted a neuroprotective effect on IS mice and on HT22 cells after induction of OGD/R. Besides, we explored whether NPGE treatment alleviated neuronal ferroptosis and ferroptosis‐mediated neuroinflammation after I/R injury. Finally, we investigated whether the NRF2/HO‐1 signaling pathway was involved in the neuroprotective effects of NPGE on IS.

## 2 MATERIALS AND METHODS

### 2.1 Drug treatment

As previously described,^16^ NPGE was obtained from the Department of Pharmacy, Renmin Hospital of Wuhan University, and was dissolved in saline as a 20 mg/mL stock solution. Brusatol (14907‐98‐3; Sigma) was dissolved in DMSO as a 5 mg/mL stock solution. All drugs were diluted to the indicated concentrations before use.

### 2.2 Animals

According to the experimental design, 160 ten‐week‐old male C57BL/6 J mice were purchased from Hunan Silaikejingda (SJA) Laboratory Animal and were individually housed in standard plastic cages. The mice were randomly assigned according to body weight, 40 in the sham group, 40 in the vehicle group, 8 in the 0.5 mg/kg NPGE treatment group, 40 in the 1 mg/kg NPGE treatment group, 8 the in 2NPGE treatment group, and 24 in the 1 mg/kg NPGE and brusatol treatment group. The mice were allowed to acclimatize to the new environment for 7 days before the experimental procedure. All animal protocols were approved by the Medical Ethics Committee of the Renmin Hospital of Wuhan University and were in complete accordance with the National Institutes of Health Guide for the Care and Use of Laboratory Animals.

### 2.3 IS model

The IS model was established under 2% isoflurane anesthesia as previously described.^18^ During surgery, the animal body temperature was maintained at 36.5°C. Left middle cerebral artery occlusion (MCAO) was induced by occlusion of the origin of the middle cerebral artery using a silk suture (Doccol Corp.) for 1.5 h. Reperfusion was initiated by removing the silk suture. Sham‐operated mice underwent the same surgical procedure but without occlusion.

### 2.4 Cell culture

The mouse hippocampal (HT22) cell line was obtained from the Zhongnan Hospital of Wuhan University. The HT22 cells were maintained in DMEM (Gibco) supplemented with 10% heat‐inactivated FBS (Gibco) and 1% penicillin–streptomycin (PS; Gibco). The cells were placed in an incubator at 37°C with 5% CO_2_.

### 2.5 OGD/R

As previously described,^19^, ^20^ HT22 cells were washed twice with PBS (Gibco) and maintained in glucose‐free DMEM (PM150270; Pricella). The cells were placed in an incubator at 1% O_2_, 5% CO_2_, and 94% N_2_ for several h. The glucose‐free DMEM was then exchanged with complete medium and culture continued under normoxic conditions (37°C, 5% CO_2_) for 12 h. The sham groups were washed twice with PBS and maintained in complete medium without oxygen deprivation.

### 2.6 Cell viability assay

Cell viability was accessed using a cell counting kit‐8 (CCK‐8; Sigma) according to the manufacturer's protocol.

### 2.7 Calcein‐AM/PI staining

According to the experimental protocol, the culture medium was removed and the cells were washed once with PBS. An appropriate volume of Calcein AM/PI (C2015S; Beyotime) detection working solution was added. Next, the cells were incubated at 37°C for 30 min in the dark, followed by observation under a fluorescence microscope.

### 2.8 Measurement of SOD Activity, MDA, Fe^2+^ and GSH

We used superoxide dismutase assay kit (Beyotime, S0109) to measure SOD activity, used lipid peroxidation MDA assay kit to measure MDA (Beyotime, S0131S), used tissue iron content assay kit to measure Fe^2+^ and used micro reduced glutathione assay kit (Solarbio, BC1175) to measure GSH. All the procedures were conducted according to the manufacturer's instructions.

### 2.9 Cerebral blood flow measurement

Mice were anesthetized with 2% isoflurane. Incised the scalp and fixed on the detection platform. Adjust the focus under the scanning probe, and imaged using a laser speckle blood flow imaging system (Simopto, Wuhan, China).

### 2.10 ROS measurement

In vivo, fresh cerebral tissue was severed at a thickness of 4.0 μm three d after surgery. Sections were incubated with 10 μM dihydroethidium (DHE; Sigma–Aldrich) for half an hour at 37°C in the dark. They were washed three times with PBS for 5 min each and then observed using a fluorescence microscope. ImageJ was used to calculate the mean fluorescence intensity of ROS–positive cells in the ischemic penumbra region. In vitro, ROS was detected by fluorescent probe H2DCFDA (Biosharp, BL714A). H2DCFDA without fluorescence can get into cells, and be hydrolyzed to generate DCFH, which cannot penetrate the cell membrane and can be oxidized into DCF by ROS. Eventually, cells were observed using a fluorescence microscope, and Image J was used to calculate the mean fluorescence intensity of ROS–positive cells.

### 2.11 Neurological score and infarct volume measurement

Three d after the surgery, the mice received a neurological deficit score, ranging from zero (no observable neurological deficits) to four (no spontaneous motor activity and loss of consciousness).^21^ The person who performed the neurological score was blinded to experimental groups. The mice were then euthanized to collect the brains. The brain sections were stained with 1.5% 2,3,5‐triphenyltetrazolium chloride (TTC) staining solution. The brain sections were scanned and the infarct volume was evaluated in a blinded manner using ImageJ, as previously described.^21^, ^22^

### 2.12 Immunofluorescence staining

Three d after the surgery, samples were collected to detect the expression of NeuN, GPX4, and NRF2. After anesthesia, the mice were perfused with pre‐cooled saline through the heart, and the brain tissues were fixed in 4% paraformaldehyde and placed at 4°C. The brains were dehydrated using 30% sucrose until they naturally sunk. The brains were cut into 5‐μm sections. After permeabilization with 0.3% Triton X for 10 min, the slices were blocked with 1% BSA for 1 h at room temperature. The blocking solution was discarded, followed by addition of a primary antibody and overnight incubation at 4°C in a wet box. The details of primary antibody was shown in Table S1. After washing thrice with PBST, Alexa488‐ or Alexa594‐conjugated antibodies (Millipore, MA, USA) were added, followed by incubation for 2 h at room temperature in the dark. After washing thrice with PBS, the cell nuclei were stained with DAPI. In each animal, three brain slices were randomly selected from the ischemic area, and positive cells were photographed in three areas of the ischemic penumbra, and the number of positively stained cells was calculated and averaged using ImageJ software. The person who analyzed the images was blinded to experimental groups.

### 2.13 Cell lysis and immunoblotting

The HT22 cells and brain tissues were lysed on ice using cold RIPA buffer, followed by addition of the protease inhibitor cocktail (Bimake). Total protein was quantified using a BCA protein assay kit (Tiangen). Equal amounts of protein samples were loaded and separated using SDS‐PAGE and transferred to nitrocellulose membranes. The primary antibodies and concentrations used for immunoblotting analysis are listed in Table S1. The primary antibodies were incubated with the samples overnight at 4°C. Horseradish peroxidase (HRP)‐conjugated goat anti‐mouse immunoglobulin or goat anti‐rabbit immunoglobulin (1:3000; Affinity) were used as secondary antibodies and incubation proceeded for 2 h at room temperature. An Immobilon Western Chemiluminescent HRP Substrate kit (Millipore) was used for detection, according to the manufacturer's instructions (Syngene). Densitometry data were analyzed using ImageJ.

### 2.14 Statistical analysis

All values represent the means ± SD. Comparisons between two groups were conducted using Student's *t*‐test. Comparisons between multiple groups were conducted using one‐way analysis of variance (ANOVA) followed by a Tukey post hoc test. Statistical significance was determined as *p* < 0.05.

## 3 RESULTS

### 3.1 The effect of NPGE treatment on mice three d after MCAO

**FIGURE 1:**
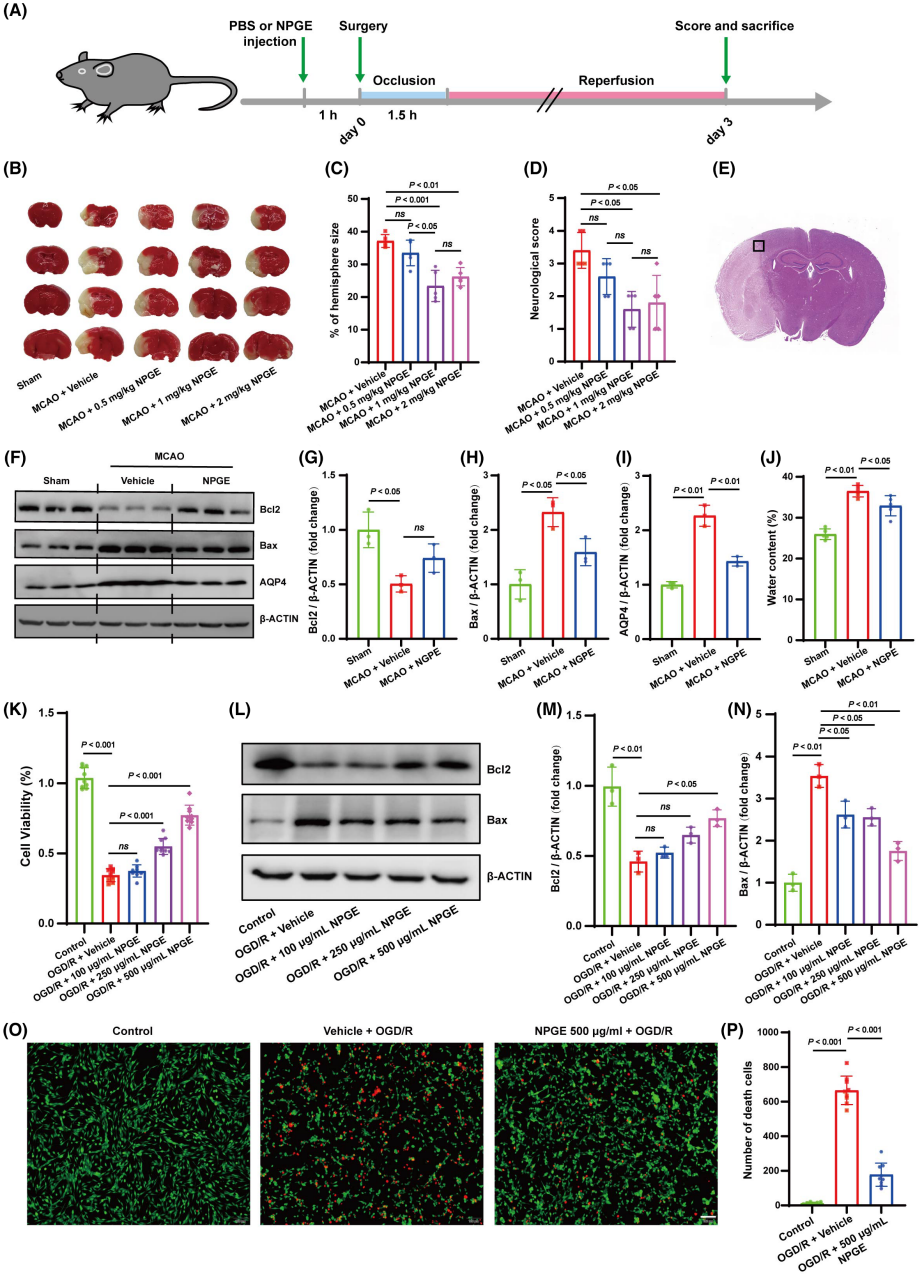
NPGE treatment alleviated I/R injury. (A) Schematic for the experimental protocol used in mice. (B, C) TTC stained brain slices showing infarct volume (white), and the infarct volume was measured in the mice treated with PBS or NPGE (0.5, 1 and 2 mg/kg) after CIRI, *n* = 5. (D) Neurological scores, *n* = 5. (E) Schematic representation of the ischemic penumbra. (F–I) Western blotting analysis of Bcl2, Bax and AQP4 in ischemic penumbra region after CIRI, *n* = 3. (J) Quantification of water content, *n* = 5. (K) Cell viability of OGD/R–induced HT22 cells treated with NPGE (0, 100, 250, 500 μg/mL), *n* = 3. (L–N) Western blotting analysis of Bcl2 and Bax in HT22 cells, *n* = 3. (O, P) Calcein–AM/PI staining of HT22 cells. Calcein–AM (green), PI (red), *n* = 3, scale bar 50 μm. *ns*, no significance.

As shown in Figure S1A, treatment with 0.5, 1, or 2 mg/kg NPGE did not result in significant body‐weight loss compared to the vehicle group, suggesting low toxicity of NPGE in vivo. To explore whether NPGE treatment had a neuroprotective effect on mice, we established an MCAO model in male C57BL/6 J mice (Figure 1A). A laser speckle flow imaging system was used to detect cerebral blood flow to determine whether the MCAO model was successful, as described^23^ (Figure S1B,C). Compared to the sham group, the mice in the experimental group showed apparent cerebral infarction and neurological deficit 3 days after MCAO (Figure 1B–D). Compared with the vehicle group, administration of 1 or 2 mg/kg NPGE reduced infarct volume and decreased the neurological deficit score (Figure 1B–D). In addition, compared with 1 mg/kg NPGE treatment, treatment with 2 mg/kg NPGE did not result in a smaller infarct volume (Figure 1B,C). Hence, we selected 1 mg/kg NPGE treatment for the subsequent in vivo experiments. After CIRI, cell death is accompanied by down‐regulation of B‐cell lymphoma‐2 (Bcl2) expression and up‐regulation of Bcl2‐associated X protein (Bax) expression.^24^ As shown in Figure 1F–H, Bcl2 expression was down‐regulated, while that of Bax was up‐regulated in the penumbra (Figure 1E). NPGE treatment down‐regulated Bax expression, although Bcl2 expression remained unchanged (Figure 1F–H).

Cerebral edema is always present after CIRI, potentially leading to increased intracranial pressure and representing the critical factor for death in patients in the acute phase of IS.^25^ Compared with the vehicle group, NPGE treatment decreased the water content and down‐regulated the expression of aquaporin 4 (AQP4), a protein thought to mediate brain edema^25^ (Figure 1F,I,J).

### 3.2 The effect of NPGE treatment on OGD/R‐induced HT22 cells

We induced OGD/R in HT22 cells to investigate whether NPGE exerted a protective effect in vitro. CCK‐8 analysis showed no significant difference when HT22 cells were treated with 100, 250, or 500 μg/mL NPGE, but treatment with 1000 μg/mL NPGE decreased cell viability (Figure S2A). Based on the obtained result, 6 h were selected to achieve OGD before R (Figure S2B). Although cell viability decreased after OGD/R, treatment with 250 or 500 μg/mL NPGE was beneficial (Figure 1K). Consistently, western blotting analysis showed that NPGE treatment (500 μg/mL) partly reversed the changes in Bcl2 and Bax (Figure 1L–N). Furthermore, calcein and PI fluorescence staining showed that application of 500 μg/mL NPGE significantly reduced the dead cell count (Figure 1O,P). These results indicate that NPGE promotes the survival of OGD/R‐induced HT22 cells. We selected 500 μg/mL NPGE for subsequent in vitro experiments.

### 3.3 The effect of NPGE treatment on oxidative stress after I/R injury

**FIGURE 2:**
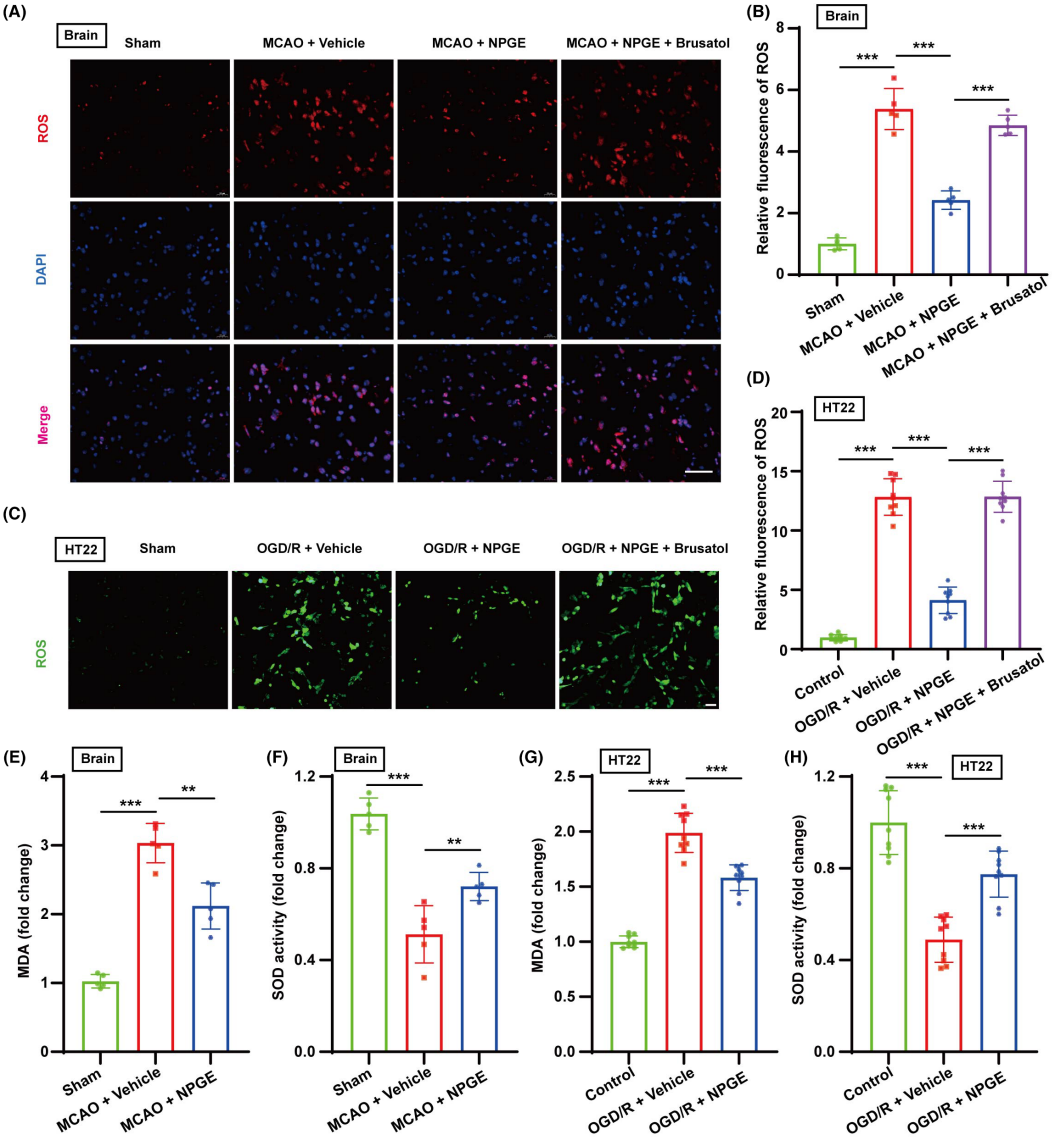
NPGE treatment alleviated oxidative stress after I/R injury. (A, B) ROS staining of mouse brains after MCAO, *n* = 5, scale bar 50 μm. (C, D) ROS staining of HT22 cells after OGD/R, *n* = 9, scale bar 50 μm. (E, F) The levels of MDA and SOD activity in the brain after CIRI, *n* = 5. (G, H) The levels of MDA and SOD activity in HT22 cells after OGD/R, *n* = 9. *p < 0.05, **p < 0.01, ***p < 0.001.

**FIGURE 3:**
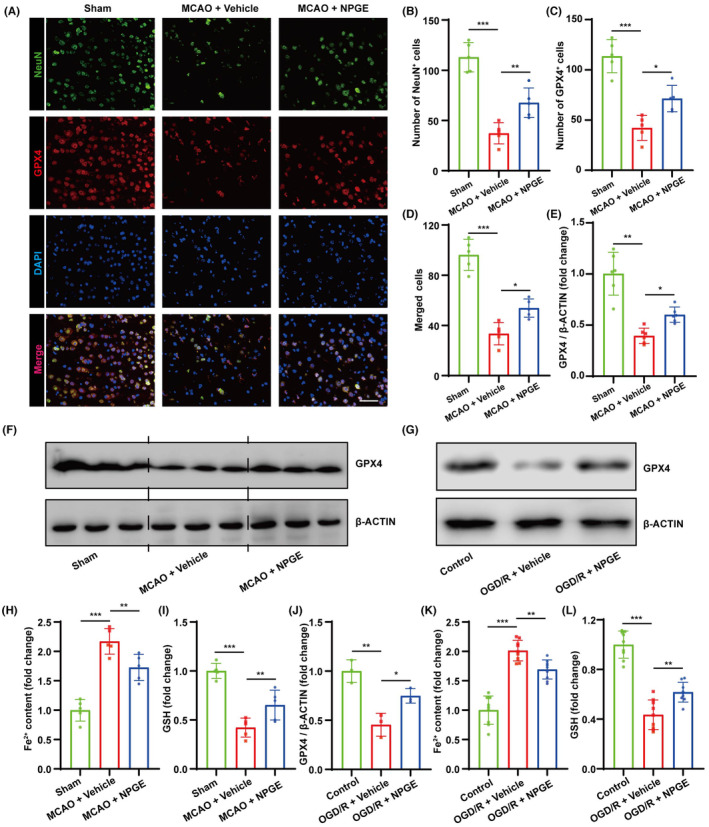
NPGE treatment alleviated neuronal ferroptosis after I/R injury. (A–D) Representative immunofluorescence images of NeuN–positive cells and GPX4–positive cells in the penumbra region after CIRI, *n* = 5, scale bar 50 μm. (E, F) Western blot analysis of GPX4 in the ischemic penumbra tissue after CIRI, *n* = 6. (G, J) Western blot analysis of GPX4 in HT22 cells after OGD/R, *n* = 3. (H, I) The levels of Fe^2+^ and GSH in the brain after CIRI, *n* = 5. (K, L) The levels of Fe^2+^ and GSH in HT22 cells after OGD/R, *n* = 9. *p < 0.05, **p < 0.01, ***p < 0.001.

*Gastrodia elata* has a strong antioxidant effect after ischemic stroke.^26^ We speculated that NPGE could inhibit oxidative stress after I/R injury. In vivo, compared with the sham group, the ROS level on the infarct side increased, SOD activity was reduced, and excess MDA content accumulated after CIRI. NPGE treatment partly reversed these changes (Figure 2A,B,E,F). The in vitro results were consistent with those observed in vivo. Compared with the vehicle group, NPGE reduced ROS and MDA levels and decreased SOD activity (Figure 3C,D,G,H). Our data thus indicate that NPGE inhibits oxidative stress after I/R injury.

### 3.4 The effect of NPGE treatment on neuronal ferroptosis after I/R injury

Oxidative stress leads to the accumulation of peroxides and disorder of the antioxidant system, which are critical ferroptosis‐inducing mechanisms. Ferroptosis is activated after CIRI and inhibiting neuronal ferroptosis can improve CIRI prognosis.^27^ Three d after MCAO, immunofluorescence staining showed that the number of NeuN‐ (a neuronal marker) and GPX4‐positive cells in the penumbra decreased, and GPX4 was mainly expressed in NeuN‐positive cells in the cerebral cortex (Figure 3A–D). In addition, western blotting analysis revealed that GPX4 expression was down‐regulated in the penumbra region (Figure 3E,F), and biochemical analysis demonstrated increased Fe^2+^ content and decreased GSH content (Figure 3H,I). These results indicate activation of neuronal ferroptosis after CIRI. NPGE treatment reversed these effects. In particular, compared with the vehicle group, NPGE treatment increased the number of NeuN‐ and GPX4‐positive cells and improved the level of GPX4 in the penumbra region (Figure 3A–C,E,F). Furthermore, application of NPGE down‐regulated Fe^2+^ content and increased GSH levels (Figure 3H,I).

Our results also revealed that NPGE treatment alleviated OGD/R‐induced ferroptosis in HT22 cells. The expression of GPX4 was down‐regulated, the level of Fe^2+^ increased, and the level of GSH decreased after OGD/R injury (Figure 3G,J–L). NPGE treatment up‐regulated the expression of GPX4, reduced Fe^2+^ content, and increased GSH content (Figure 3G,J–L). These results demonstrate that NPGE inhibits neuronal ferroptosis induced by I/R injury both in vivo and in vitro.

### 3.5 The effect of NPGE treatment on ferroptosis‐induced neuroinflammation

**FIGURE 4:**
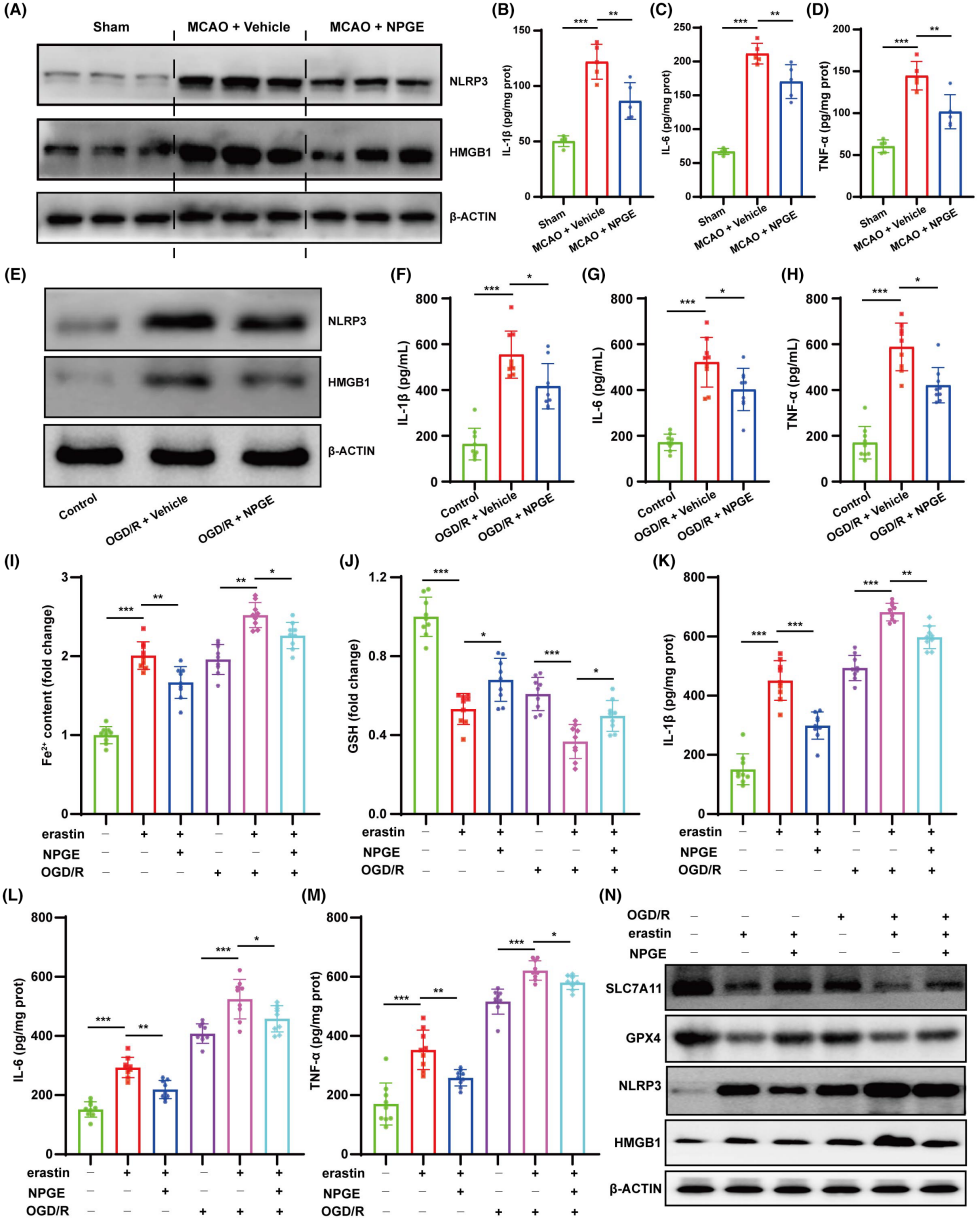
NPGE treatment attenuated ferroptosis–related neuroinflammation after I/R injury. (A) Western blotting analysis of NLRP3 and HMGB1 in the ischemic penumbra tissue after CIRI, *n* = 6. (B–D) The levels of IL–1β, IL–6 and TNF–α in the ischemic penumbra tissue, *n* = 5. (E) Western blotting analysis of NLRP3 and HMGB1 in HT22 cells after OGD/R, *n* = 3. (F–H) The levels of IL–1β, IL–6 and TNF–α in HT22 cells after OGD/R, *n* = 9. (I, J) The levels of Fe^2+^ and GSH in erastin‐exposed HT22 cells, *n* = 9. (K–M) The levels of IL–1β, IL–6 and TNF–α in erastin‐exposed HT22 cells, *n* = 9. (N) Western blot analysis of SLC7A11, GPX4, NLRP3 and HMGB1 in erastin‐exposed HT22 cells, *n* = 3. *p < 0.05, **p < 0.01, ***p < 0.001.

The crosstalk between ferroptosis and neuroinflammation affects CIRI outcome.^28^ In mice, our results showed that the expression of NLRP3, HMGB1, IL‐1β, IL‐6, and TNFα increased after CIRI (Figure 4A–D and Figure S3A,B). NPGE treatment down‐regulated the expression of NLRP3 and HMGB1 and decreased the levels of IL‐1β, IL‐6, and TNFα (Figure 4A–D and Figure S3A,B). In vitro, OGD/R injury induced inflammation in HT22 cells. Compared with the control group, NPGE treatment decreased NLRP3, HMGB1, IL‐1β, IL‐6, and TNF‐α levels (Figure 4E–H and Figure S3C,D). Hence, our data demonstrate that NPGE treatment can alleviate neuroinflammation after I/R injury.

It has been reported that ferroptosis can induce neuroinflammation.^29^ To explore whether NPGE regulated neuroinflammation by inhibiting ferroptosis, we co‐treated HT22 cells with NPGE and erastin, a ferroptosis inducer. Interestingly, treatment with erastin activated ferroptosis and inflammation in HT22 cells. It increased the levels of Fe^2+^ and decreased those of GSH, SLC7A11, and GPX4 (Figure 4I,N and Figure S3E,F). In addition, erastin treatment up‐regulated the levels of NLRP3, HMGB1, IL‐1β, IL‐6, and TNFα (Figure 4K–N and Figure S3G,H). Its application aggravated ferroptosis and neuroinflammation in OGD/R‐induced HT22 cells; it increased the levels of Fe^2+^, NLRP3, IL‐1β, IL‐6, and TNFα and decreased those of GSH, SLC7A11, and GPX4 (Figure 4I–N and Figure S3E–H). Application of NPGE partly alleviated these effects. These data suggest that NPGE can alleviate neuronal ferroptosis‐mediated neuroinflammation after I/R injury.

### 3.6 The effect of NPGE treatment on the NRF2/HO‐1 signaling pathway after I/R injury

**FIGURE 5:**
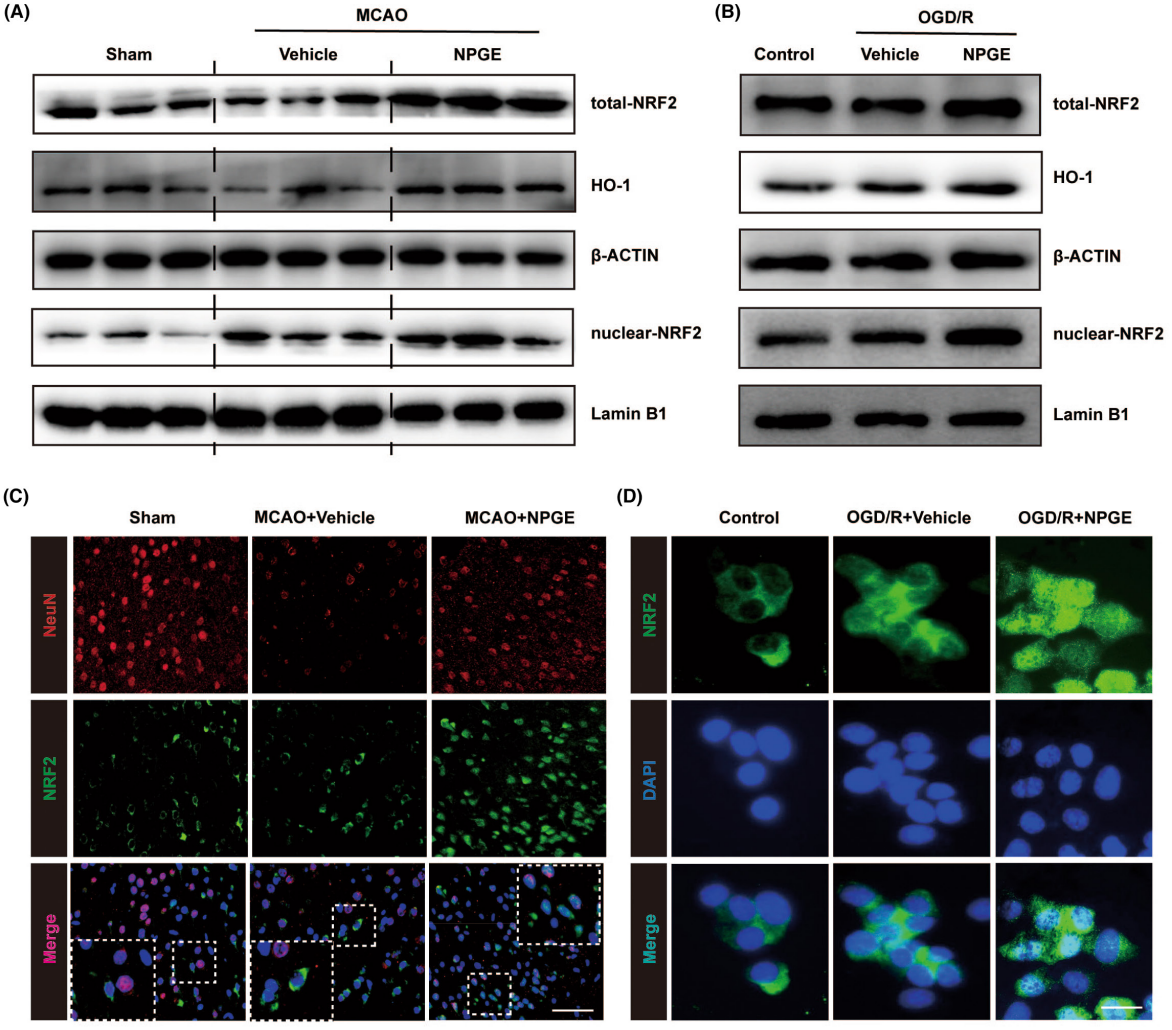
NPGE treatment activated NRF2/HO–1 signaling pathway after I/R injury. (A) Western blotting analysis of total–NRF2, HO–1 and nuclear–NRF2 in the ischemic penumbra tissue after CIRI, *n* = 6. (B) Western blot analysis of total–NRF2, HO–1 and nuclear–NRF2 in HT22 cells after OGD/R, *n* = 3. (C) Representative immunofluorescence images of NeuN–positive cells and NRF2–positive cells in the ischemic penumbra tissue after CIRI, *n* = 5, scale 40 μm. (D) Representative immunofluorescence images of NRF2 in HT22 cells, *n* = 5, scale 10 μm. *p < 0.05, **p < 0.01, ***p < 0.001.

We performed western blotting analysis to reveal the underlying molecular mechanism of NPGE action on I/R injury. As shown in Figure 5A and Figure S4A,B, NPGE up‐regulated the expression of total NRF2 and HO‐1 after CIRI, compared with the vehicle group. The same results were observed in OGD/R‐induced HT22 cells (Figure 5B and Figure S4D,E). Upon activation, NRF2 translocates to the nucleus to initiate gene transcription.^30^ Western blotting analysis demonstrated that NPGE treatment increased the levels of nuclear NRF2 in IS mice and in HT22 cells after OGD/R induction (Figure 5A,B and Figure S4C,F), indicating that NPGE may promote NRF2 nuclear translocation. Based on these findings, we performed immunofluorescence staining to reveal potential changes in the cellular localization of NRF2. Our results showed that NRF2 was mainly expressed in the cytoplasm in neurons in the sham and vehicle groups, while NPGE treatment increased the levels of nuclear NRF2 (Figure 5C,D). Therefore, NPGE treatment activates the NRF2/HO‐1 signaling pathway and promotes the nuclear translocation of NRF2 after I/R injury.

### 3.7 The effect of brusatol on the neuroprotective capacity of NPGE after I/R injury

**FIGURE 6:**
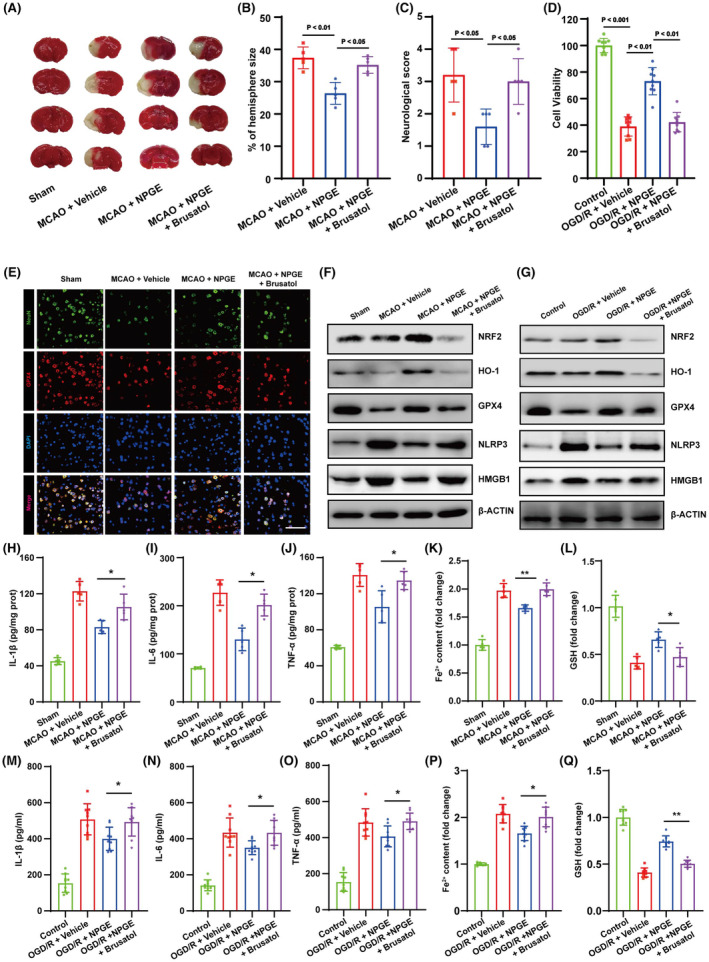
Brusatol treatment abolished the neuroprotective effect of NPGE. (A, B) TTC stained brain slices showing infarct volume (white), and the infarct volume was measured in the mice after CIRI, *n* = 5. (C) Neurological scores, *n* = 5. (D) Cell viability of HT22 cells after OGD/R, *n* = 9. (E) Representative immunofluorescence images of NeuN–positive cells and GPX4–positive cells in the penumbra region after CIRI, *n* = 5, scale bar, 50 μm. (F) Western blotting analysis of NRF2, HO–1, GPX4, NLRP3 and HMGB1 in the ischemic penumbra tissue after CIRI, *n* = 5. (G) Western blotting analysis of NRF2, HO–1, GPX4 NLRP3 and HMGB1 in HT22 cells after OGD/R, *n* = 3. (H–J) The levels of IL–1β, IL–6 and TNF–α in the brain after CIRI, *n* = 5. (K, L) The levels of Fe^2+^ and GSH in the brain after CIRI, *n* = 5. (M–O) The levels of IL–1β, IL–6 and TNF–α in HT22 cells after OGD/R, *n* = 3. (P, Q) The levels of Fe^2+^ and GSH in HT22 cells after OGD/R, *n* = 3. *p < 0.05, **p < 0.01.

We used brusatol, an NRF2 inhibitor, to determine whether NPGE exerted neuroprotective effects via the NRF2/HO‐1 signaling pathway. As shown in Figure 6A, we co‐treated mice with brusatol and NPGE, and TTC staining demonstrated that the addition of the NRF2 inhibitor increased the infarct volume compared with the NPGE group (Figure 6A,B). In addition, brusatol increased the neurological score (Figure 6C). In vitro, co‐treatment with NPGE and brusatol lowered the viability of HT22 cells after OGD/R (Figure 6D). These results indicate that application of the NRF2 inhibitor abolishes the neuroprotective capacity of NPGE after I/R injury.

### 3.8 The effect of brusatol on anti‐ferroptotic and anti‐neuroinflammatory effects of NPGE treatment after I/R injury

The NRF2/HO‐1 signaling pathway exerts a protective role in I/R injury by regulating ferroptosis.^31^ Therefore, we investigated whether NPGE mediated neuronal ferroptosis and neuroinflammation through NRF2/HO‐1 signaling. Immunofluorescence staining revealed that brusatol treatment reduced the NeuN‐ and GPX4‐positive cells in the penumbra region (Figure 6E and Figure S5A,B) after CIRI. Moreover, western blotting analysis demonstrated that the NRF2 inhibitor decreased the expression of NRF2, HO‐1, and GPX4. (Figure 6F and Figure S5C–E). Brusatol treatment also increased the levels of ROS and Fe^2+^ and decreased GSH levels compared with the NPGE group after CIRI (Figures 2A,B and 6K,L). These data suggest that brusatol inhibits the anti‐ferroptotic activity of NPGE. Further, the results showed that treatment with brusatol up‐regulated the levels of NLRP3, HMGB1, IL‐1β, IL‐6, and TNFα, indicating successful inhibition of the anti‐neuroinflammatory activity of NPGE after CIRI (Figure 6F,H–L and Figure S5F,G).

In vitro, western blotting indicated that the NRF2 inhibitor treatment down‐regulated the NRF2, HO‐1, and GPX4 and upregulated the NLRP3 and HMGB1 levels in HT22 cells after OGD/R, compared with the NPGE treatment alone (Figure 6G,M–Q and Figure S5H–L). Moreover, the NRF2 inhibitor increased the production of ROS, Fe^2+^, IL‐1β, IL‐6, and TNF‐α and decreased that of GSH (Figures 2C,D and 6M–O). Our data demonstrate that brusatol treatment abolishes the anti‐ferroptotic and anti‐neuroinflammatory effects of NPGE after I/R injury.

## 4 DISCUSSION

Ischemic stroke, caused by arterial embolism, is a common insult to the nervous system, and its high fatality and disability rates pose a massive threat to human health.^1^ Drug development to improve IS outcomes is thus of great value. Increasing evidence shows that targeting ferroptosis and neuroinflammation are novel and promising therapeutic strategies for IS treatment.^32^, ^33^ In this study, we investigated the effect of NPGE on CIRI prognosis and revealed the underlying mechanisms. Our data demonstrated that NPGE decreased the infarct volume, neurological deficits, and cerebral edema 3 days after MCAO and improved survival in HT22 cells after OGD/R. Furthermore, NPGE inhibited oxidative stress in IS mice and OGD/R‐induced HT22 cells. In addition, NPGE alleviated neuronal ferroptosis‐mediated neuroinflammation and activated the NRF2/HO‐1 signaling pathway. Lastly, using the NRF2 inhibitor brusatol, we found that NRF2/HO‐1 signaling mediated the anti‐ferroptotic and anti‐neuroinflammatory effects of NPGE after I/R injury.

Multiple programmed cell death modalities are involved in neurological death after CIRI, including ferroptosis.^32^ After CIRI, the blood–brain barrier is disrupted and large amounts of free iron and ferritin enter brain tissue. The acidic environment caused by the ischemia can promote the dissociation of Fe^2+^ from transferrin, leading to increased extracellular Fe^2+^ levels, which in turn promotes increased iron uptake by neurons.^5^ Excess Fe^2+^ increases ROS production via the Fenton reaction and promotes lipid peroxidation, leading to the accumulation of lipid peroxides.^3^ GSH, an important antioxidant, is reduced to GSSC and converts lipid peroxides to non‐toxic lipid alcohols through the action of the core protein GPX4.^34^ After CIRI, the accumulation of lipid peroxides consumes a large amount of GSH, while GPX4 expression and activity are reduced, resulting in a serious imbalance in the antioxidant system.^5^ The massive accumulation of peroxides and the severe deficiency in antioxidant capacity activate ferroptosis, eventually causing cellular damage and even death.^35^ Our results showed that ROS, MDA, and Fe^2+^ levels increased, while GSH, GPX4, and SOD activity decreased in IS mice and HT22 cells after OGD/R, indicating activation of ferroptosis, in line with other studies.^27^, ^36^, ^37^ Targeting ferroptosis is beneficial for improving CIRI prognosis.^3^ Iron ion accumulation is an important factor driving ferroptosis, and iron chelators are effective in reducing free radical production and delaying neuronal death after IS.^38^ The herbal compound Naotaifang extract reduces Fe^2+^ accumulation by reversing the levels of transferrin receptor 1 and divalent metal transporter 1 and improves the neurological and behavioral scores in MCAO rats.^39^ Lipid peroxide accumulation is an important cause of cellular damage. The activation of cytoplasmic phospholipase A2α (CPLA2α) after loss of cerebral ischemia–reperfusion promotes lipid peroxidation of polyunsaturated fatty acids causing lipid peroxide accumulation, while CPLA2α knockdown significantly reduces lipid peroxidation and decreases the volume of cerebral infarction.^40^ Enhancement of the antioxidant system is beneficial against ferroptosis. Studies have shown that supplementation with selenocysteine can increase GSH levels and promote GPX4 expression to attenuate CIRI.^41^ In our study, NPGE treatment was effective in reducing cortical ROS, Fe^2+^, and MDA levels, increasing GSH levels and SOD activity, and upregulating GPX4 expression in vivo and in vitro. These results suggest that NPGE can effectively reduce CIRI‐induced neuronal ferroptosis.

Although ferroptosis and neuroinflammation can independently influence the prognosis of stroke,^4^, ^5^, ^42^ recent studies have shown that ferroptosis and neuroinflammation are interrelated.^43^, ^44^ Currently, the principal concept is that ferroptosis promotes neuroinflammation.^28^, ^44^ Mechanistically, a decrease in GPX4 levels leads to an increase in ROS production, and ROS accumulation can activate the NF‐κB pathway, promote the expression of proinflammatory factors, and activate microglia/macrophages to a proinflammatory phenotype.^28^ In addition, ferroptosis is linked to arachidic acid metabolism and eicosanoid biosynthesis, which affect the expression of PTGS2 that regulates the secretion of inflammatory factors.^45^ Moreover, ferroptosis induces the release of damage‐associated molecular patterns, such as HMGB1, which are known to amplify the inflammatory response.^28^ Inflammatory responses may also promote ferroptosis. Proinflammatory factors can reduce GPX4 levels and trigger ferroptosis in tumor cells, and NLRP3 knockdown in mice can improve the levels of GPX4 in the brain.^37^, ^46^ We found that NPGE can alleviate neuroinflammation after I/R injury, but whether the anti‐ferroptotic and anti‐inflammatory effects of NPGE are linked remains unknown. Recently, it was reported that neuronal ferroptosis can activate inflammatory responses in HT22 cells.^29^ Therefore, we investigated whether NPGE could alleviate ferroptosis‐mediated neuroinflammation. Interestingly, NPGE did alleviate erastin‐induced inflammation in HT22 cells. Furthermore, erastin aggravated OGD/R‐induced ferroptosis and neuroinflammation, but NPGE alleviated ferroptosis‐mediated neuroinflammation in OGD/R‐induced HT22 cells. These results may indicate that NPGE reduces ferroptosis‐mediated neuroinflammation after I/R injury. The crosstalk between neuronal ferroptosis and neuroinflammation influences the prognosis after CIRI, yet the organism is complex and multi‐systems interconnected, and even though we have found that NPGE modulates ferroptosis‐induced neuroinflammation in vitro experiments, we nonetheless lack sufficient evidence to demonstrate whether NPGE attenuates neuroinflammation through inhibition of ferroptosis after MCAO, or whether they act separately, or both of them at the same time. Hence, further investigations and additional evidence from in vivo experiments are required.

NRF2 is a protective transcription factor that is widely expressed in various cell types and regulates the expression of several protective genes.^47^, ^48^ Under physiological conditions, NRF2 exists as a complex after synthesis and is quickly degraded by the proteasome, to maintain a low abundance and activity.^47^ NRF2 is reported to regulate ferroptosis.^31^ NRF2 dissociates from Keap1 after activation and enters the nucleus to transcriptionally regulate the expression of GPX4.^31^ In addition, NRF2 can regulate inflammatory responses.^49^ NRF2 activation can induce the expression of HO‐1. On the one hand, HO‐1 can directly regulate the expression of proinflammatory factors; on the other hand, HO‐1 can catalyze the decomposition of heme to generate CO, which can inhibit the expression of the classical inflammatory regulator NF‐κB, thereby inhibiting the proinflammatory signal.^48^ We found that NPGE up‐regulated the expression of NRF2 and HO‐1 and promoted the translocation of NRF2 into the nucleus. Therefore, we hypothesize that NPGE exerts anti‐ferroptotic and anti‐inflammatory activities by regulating NRF2. Interestingly, brusatol, an NRF2 inhibitor, abolished the protective effects of NPGE on neuronal ferroptosis and neuroinflammation, suggesting that NRF2/HO‐1 signaling modulates the anti‐ferroptotic and anti‐neuroinflammatory effects of NPGE.

In conclusion, our study indicates that NPGE alleviates CIRI by attenuating ferroptosis‐mediated neuroinflammation via the NRF2/HO‐1 signaling pathway, which may provide a new choice for IS therapy.

## FUNDING INFORMATION

This work was supported by the National Natural Science Foundation of China (No. 82171336, 82371346 and 81870939 to Xiaoxing Xiong, 82271370 to Lijuan Gu, and 81503051 to Peng Ye).

## CONFLICT OF INTEREST STATEMENT

The authors declare no conflict of interest.

## Supporting information


Data S1.

Data S2.

## Data Availability

Research data are not shared.
