# Supplementary material for: Neutral polysaccharide from Gastrodia elata alleviates cerebral ischemia–reperfusion injury by inhibiting ferroptosis‐mediated neuroinflammation via the NRF2/HO‐1 signaling pathway
Source: CNS Neurosci Ther. 2023 Sep 26;30(3):e14456. doi: 10.1111/cns.14456 (PMC10916450; doi:10.1111/cns.14456)
Supplement: Supplementary file 1 — Data S1. [file CNS-30-e14456-s001.docx]

**Supplemental material**

**Supplemental Table 1**. Primary antibodies used in this study

| Reagents | Source | Catalog number | Application details |
| --- | --- | --- | --- |
| NeuN | abcam | ab104224 | IF 1:200 |
| GPX4 | abcam | ab125066 | IF 1:200; IB 1:1000 |
| NRF2 | Cell Signaling Technology | 12721 | IF 1:200; IB 1:1000 |
| NLRP3 | abcam | ab263899 | IB 1:1000 |
| HMGB1 | abcam | ab18256 | IB 1:1000 |
| Bcl2 | Beyotime | AF6285 | IB 1:500 |
| Bax | Beyotime | AF0057 | IB 1:500 |
| AQP4 | abcam | ab259318 | IB 1:1000 |
| HO-1 | Cell Signaling Technology | 86806 | IB 1:1000 |
| β-ACTIN | Cell Signaling Technology | 3700 | IB 1:1000 |
| SLC7A11 | Abmart | T57046 | IB 1:1000 |
| Lamin B1 | abcam | ab16048 | IB 1:1000 |

Supplemental Figures


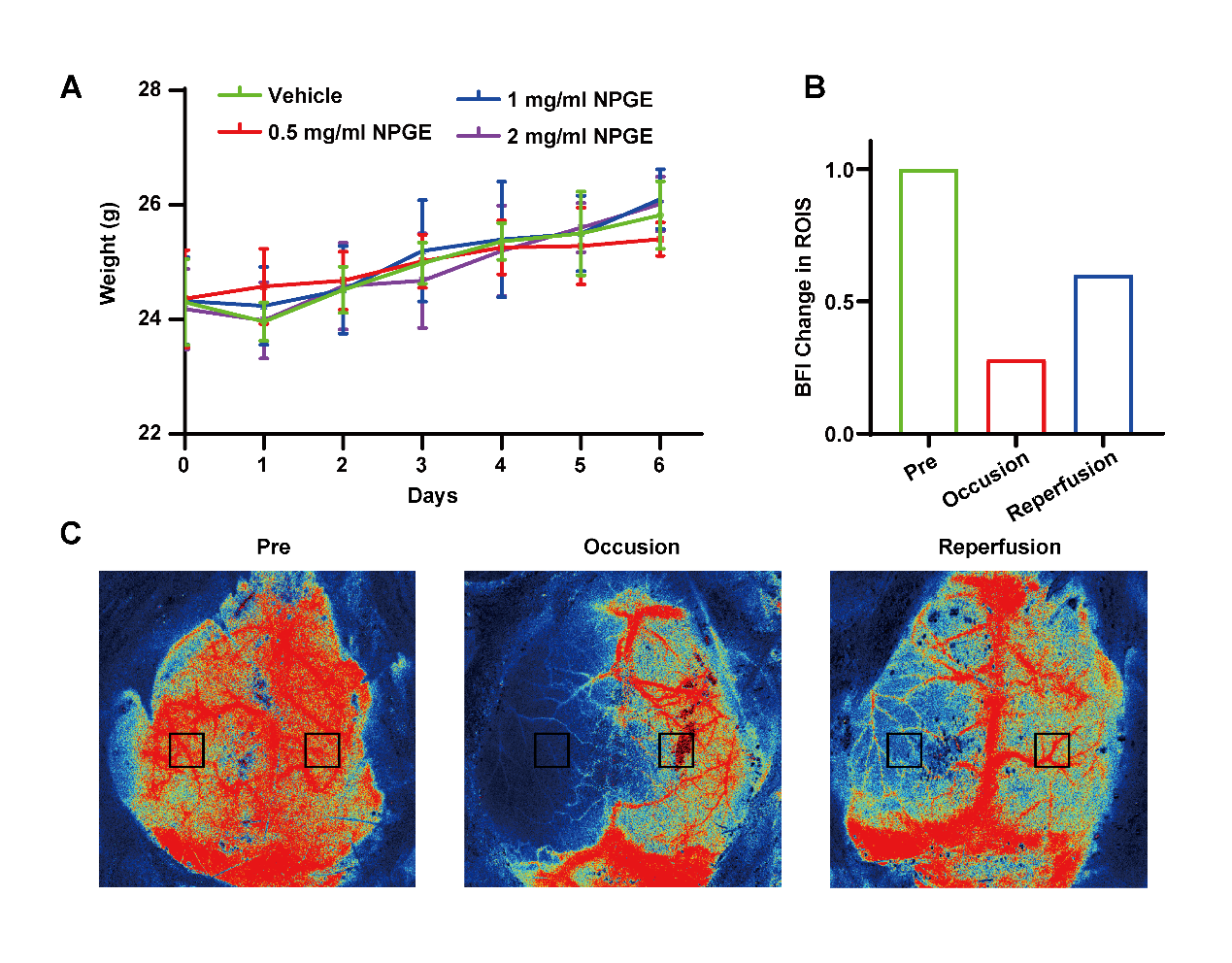


**Supplemental Figure 1. (A)** The body weight of mice treated with NPGE, n = 5. (**B,C**) The cerebral blood flow of mice. ROIs regions of interest. BFI blood flow index.


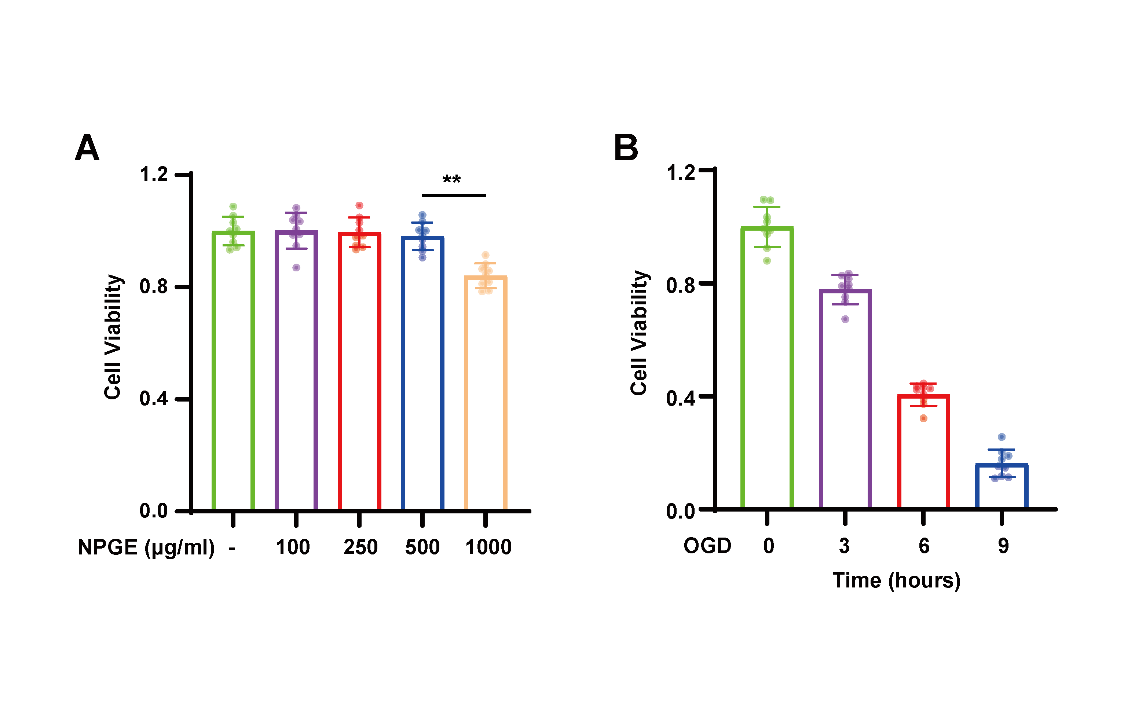
 **Supplemental Figure 2. (A)** Cell viability of HT22 cells treated with NPGE (0, 100, 250, 500 and 1,000 µg/mL), n = 9. **(B)** Cell viability of HT22 cells with different time for OGD before reperfusion, n = 9. ** *P* < 0.01.


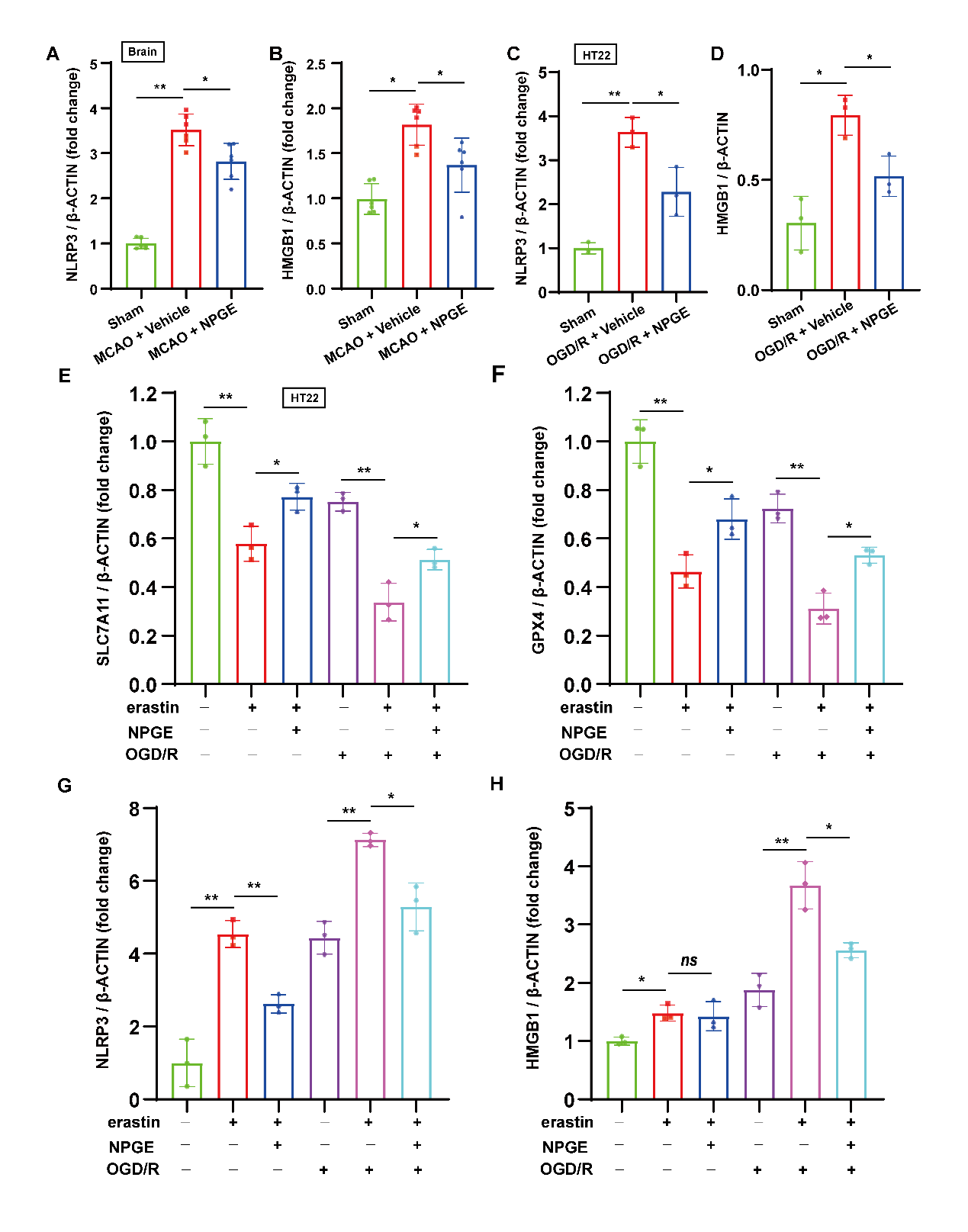


**Supplemental Figure 3. (A,B)** Quantitative analysis of the protein levels of NLRP3 and HMGB1 in ischemic penumbra region after CIRI, n = 6. **(C,D)** Quantitative analysis of the protein levels of NLRP3 and HMGB1 in OGD/R-induced HT22 cells, n = 3. **(E-H)** Quantitative analysis of the protein levels of SLC7A11, GPX4, NLRP3 and HMGB1 in erastin-induced HT22 cells, n = 3. **P* < 0.05, ** *P* < 0.01. *ns* no significance.


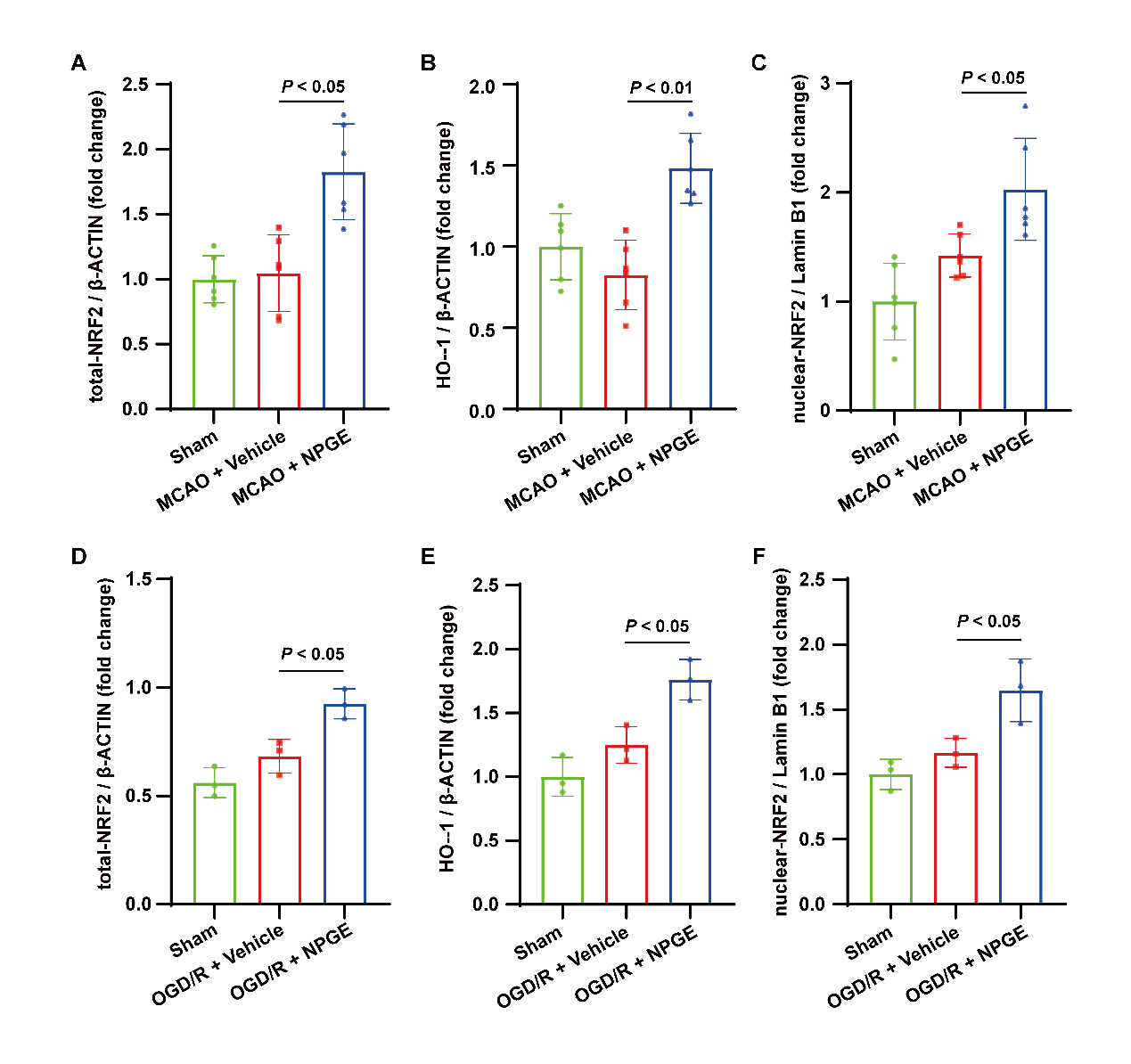


**Supplemental Figure 4. (A-C)** Quantitative analysis of the protein levels of total-NRF2, HO-1 and nuclear-NRF2 in ischemic penumbra region after CIRI, n = 6. **(D-F)** Quantitative analysis of the protein levels of total-NRF2, HO-1 and nuclear-NRF2 in OGD/R-induced HT22 cells, n = 3.


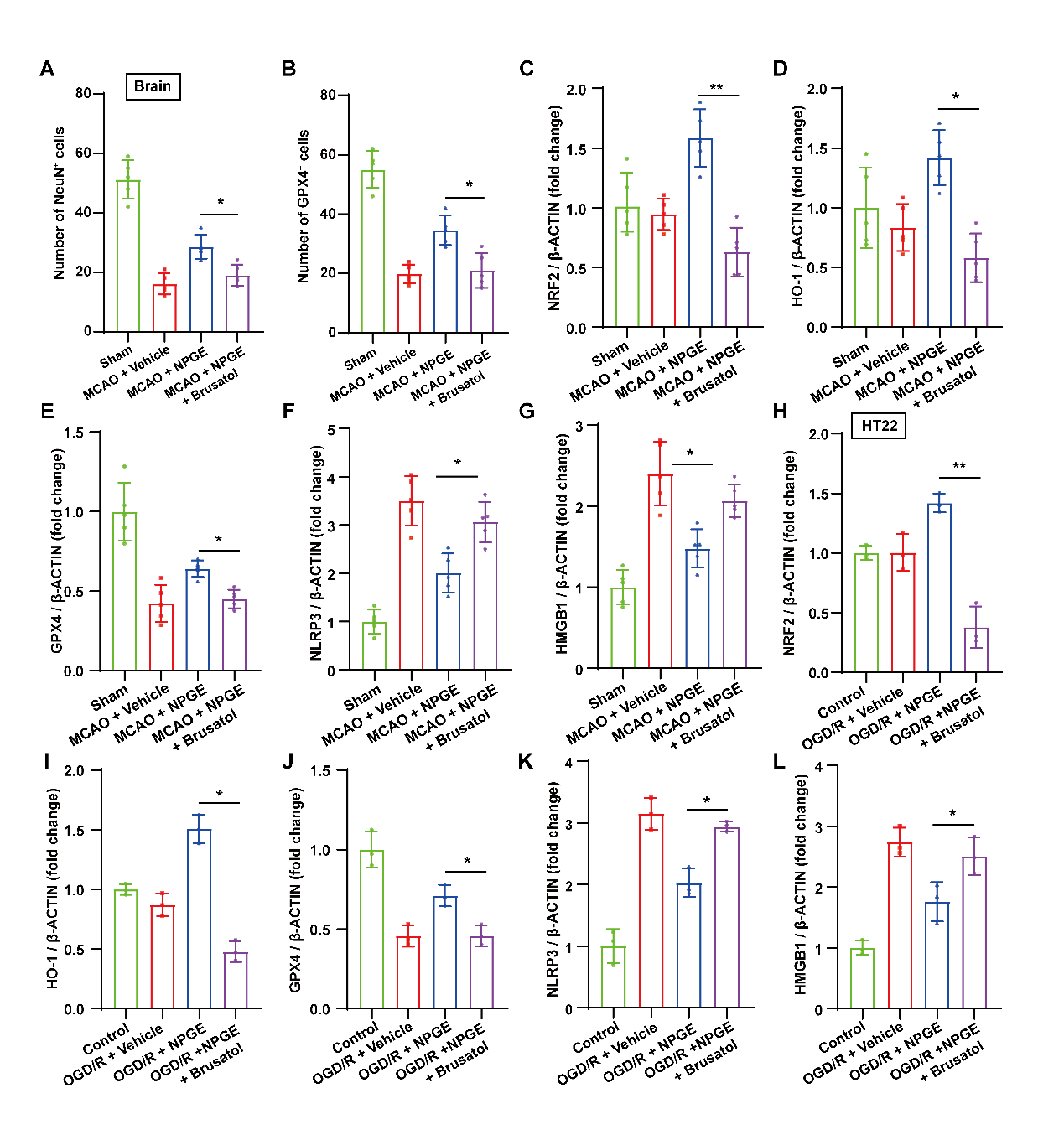


**Supplemental Figure 5. (A-B)** Quantification of NeuN- and NLRP3-positive cells in the ischemic penumbra after CIRI, n = 6. **(C-G)** Quantitative analysis of the protein levels of NRF2, HO-1, GPX4, NLRP3 and HMGB1 in ischemic penumbra region after CIRI, n = 6.Quantitative analysis of the protein levels of NRF2, HO-1, GPX4, NLRP3 and HMGB1 in OGD/R-induced HT22 cells, n = 3. **P* < 0.05, ** *P* < 0.01.
