# Supplementary figures and images for: Neutral polysaccharide from Gastrodia elata alleviates cerebral ischemia–reperfusion injury by inhibiting ferroptosis‐mediated neuroinflammation via the NRF2/HO‐1 signaling pathway
Source: CNS Neurosci Ther. 2023 Sep 26;30(3):e14456. doi: 10.1111/cns.14456 (PMC10916450; doi:10.1111/cns.14456)

Figure 1F

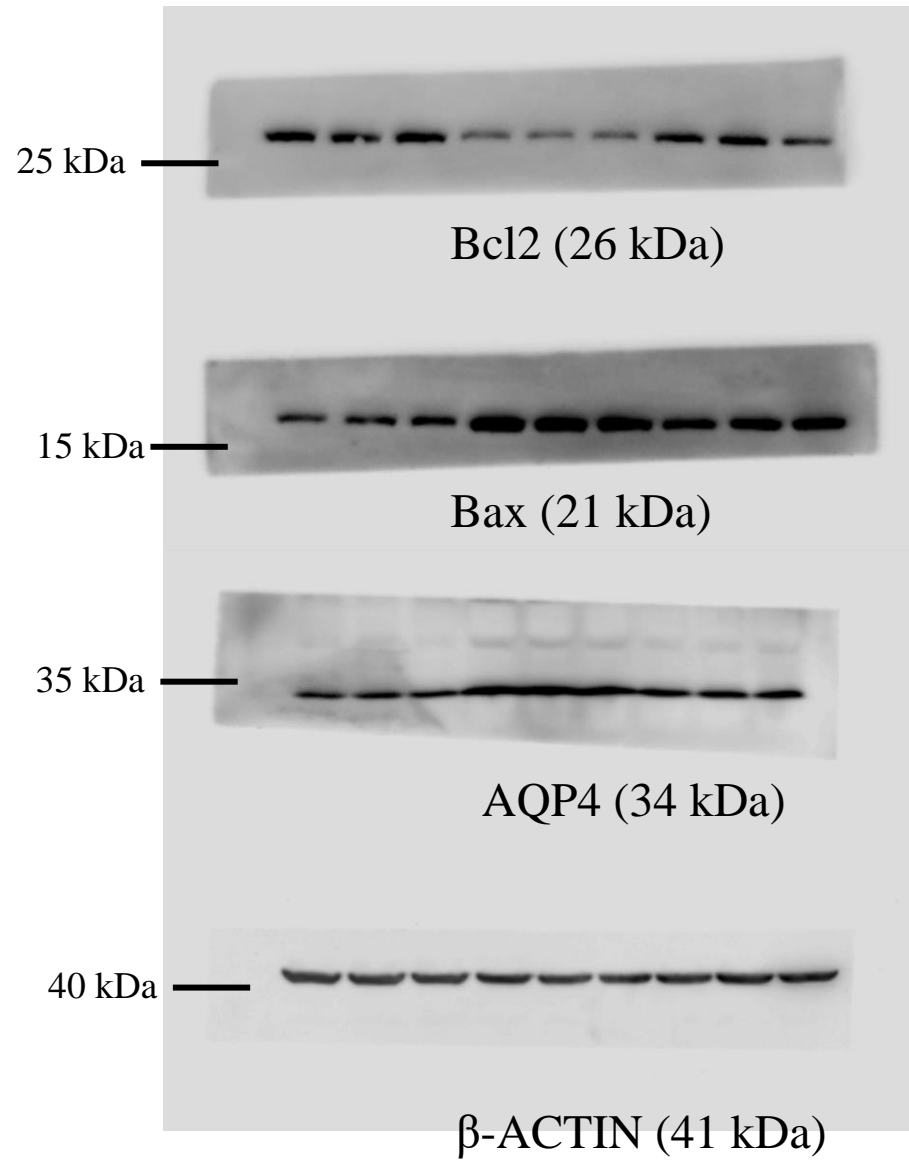

Figure 1L

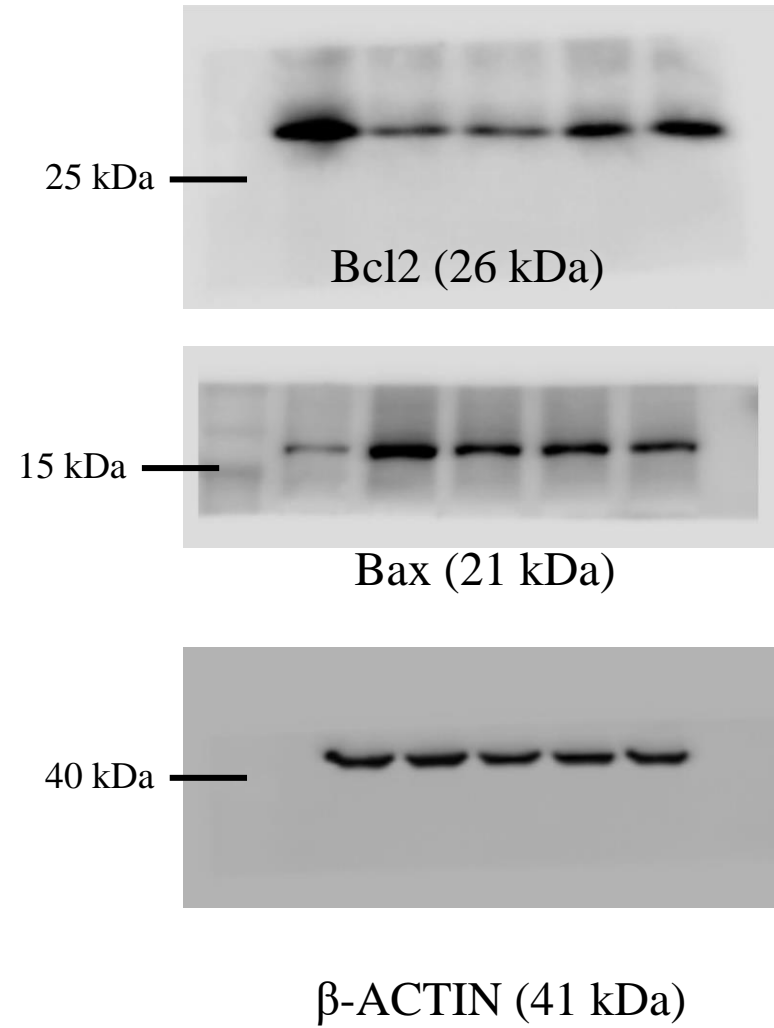

Figure 3F

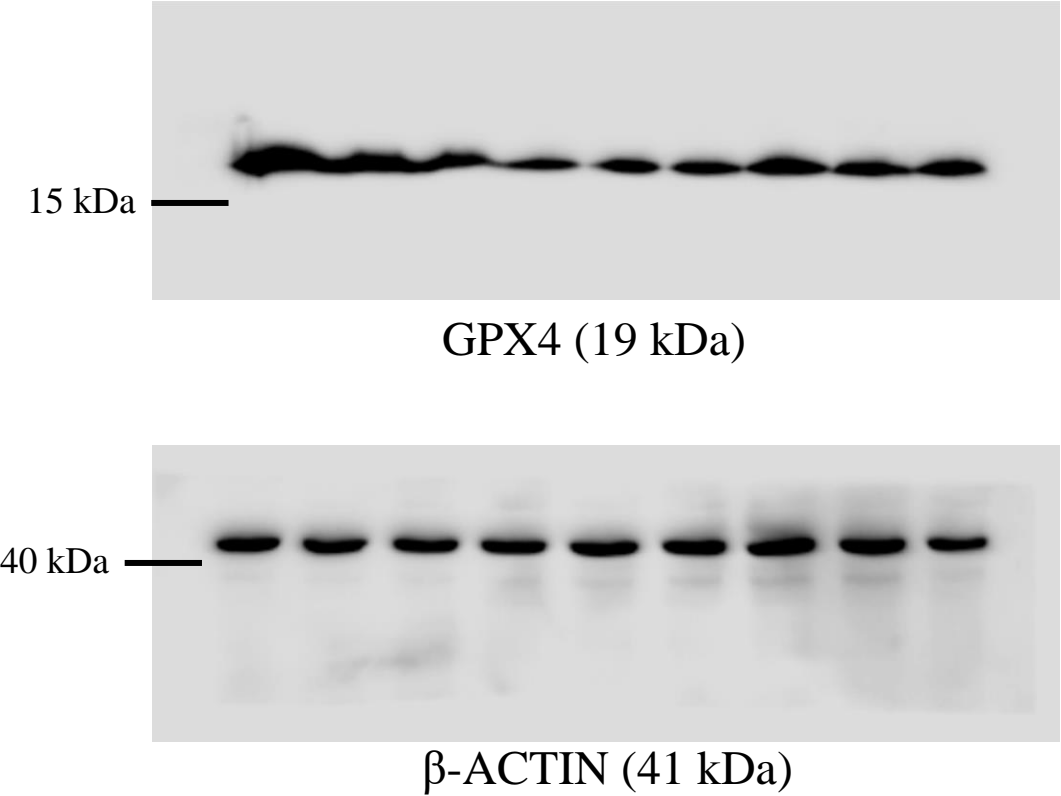

Figure 3G

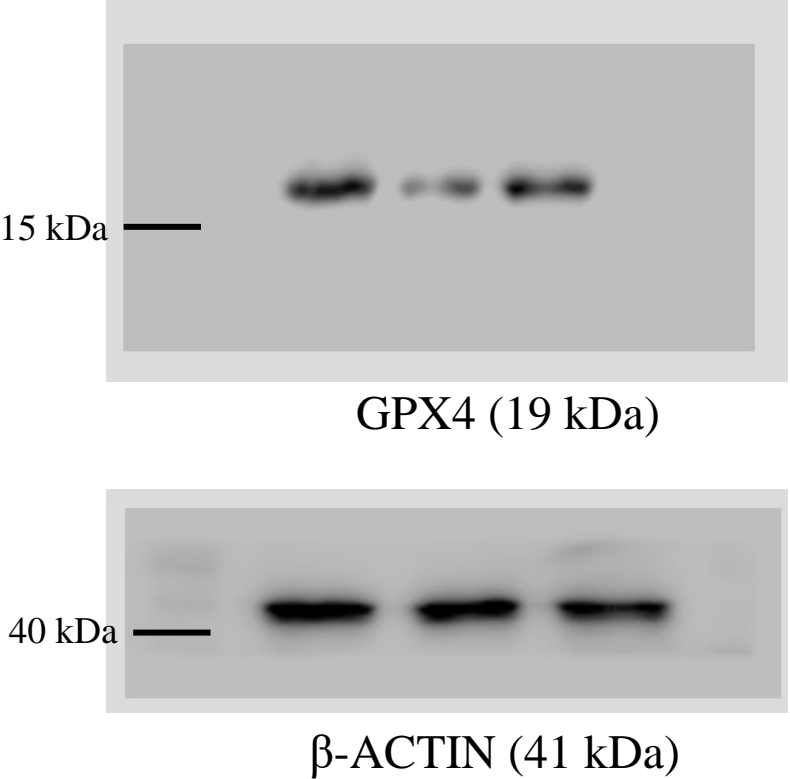

Figure 4A

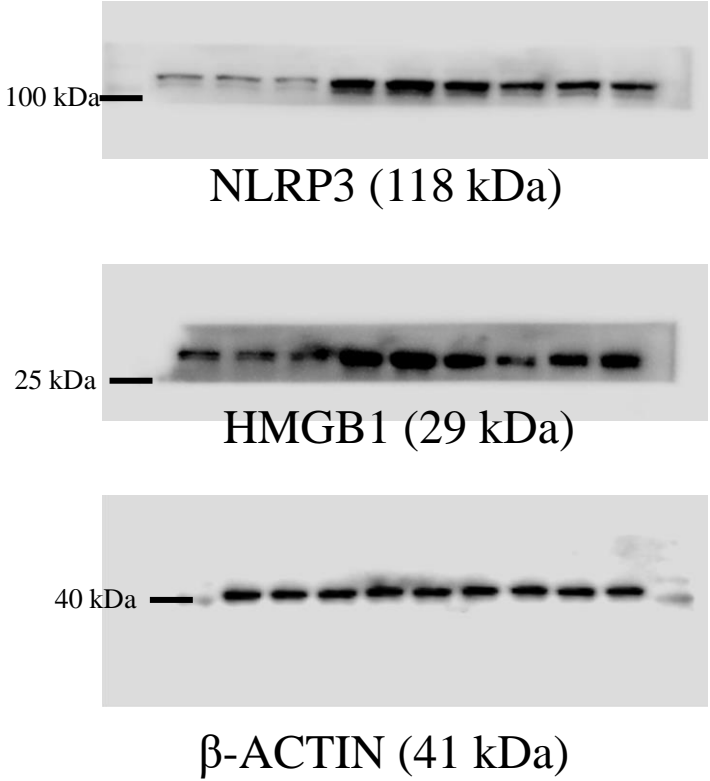

Figure 4E

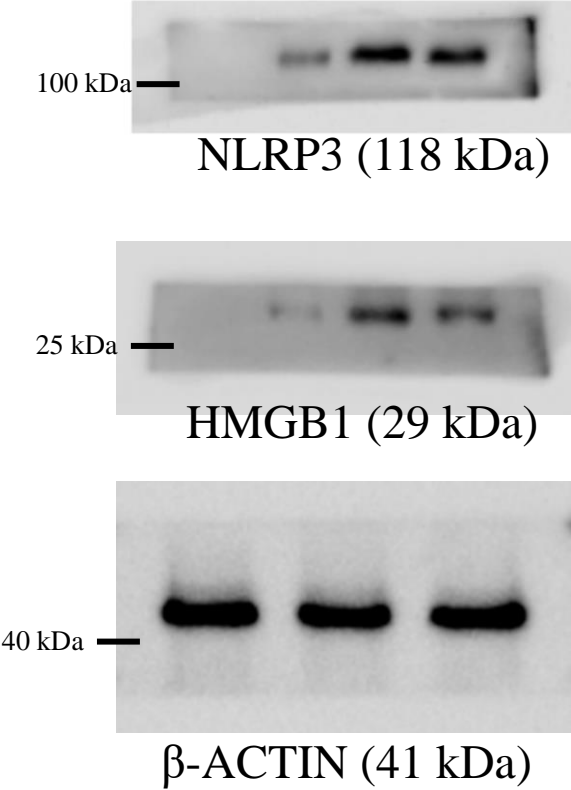

Figure 4N

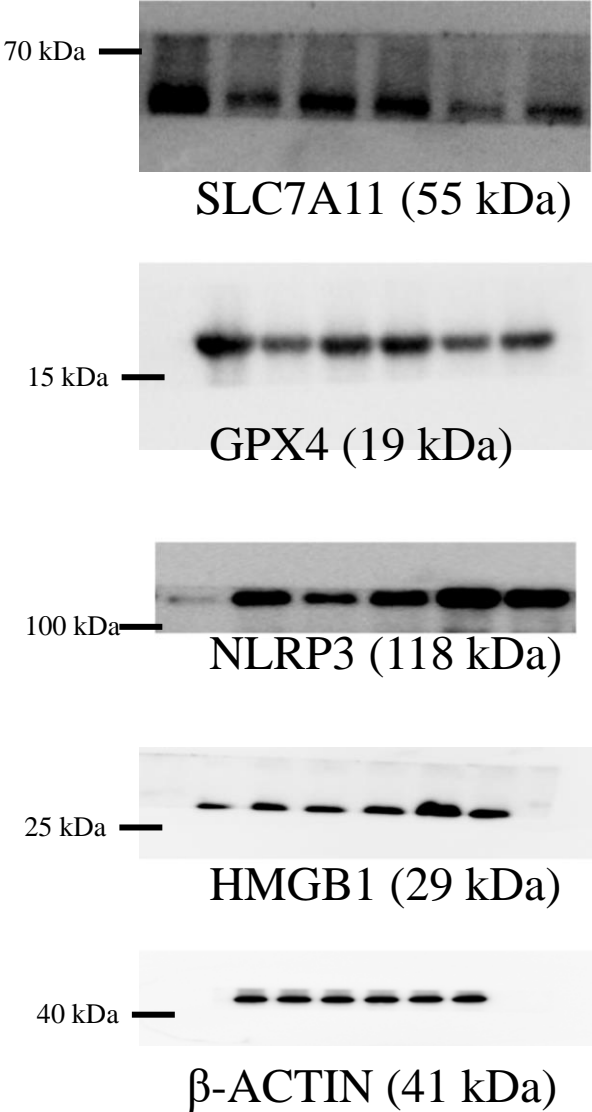

Figure 5A

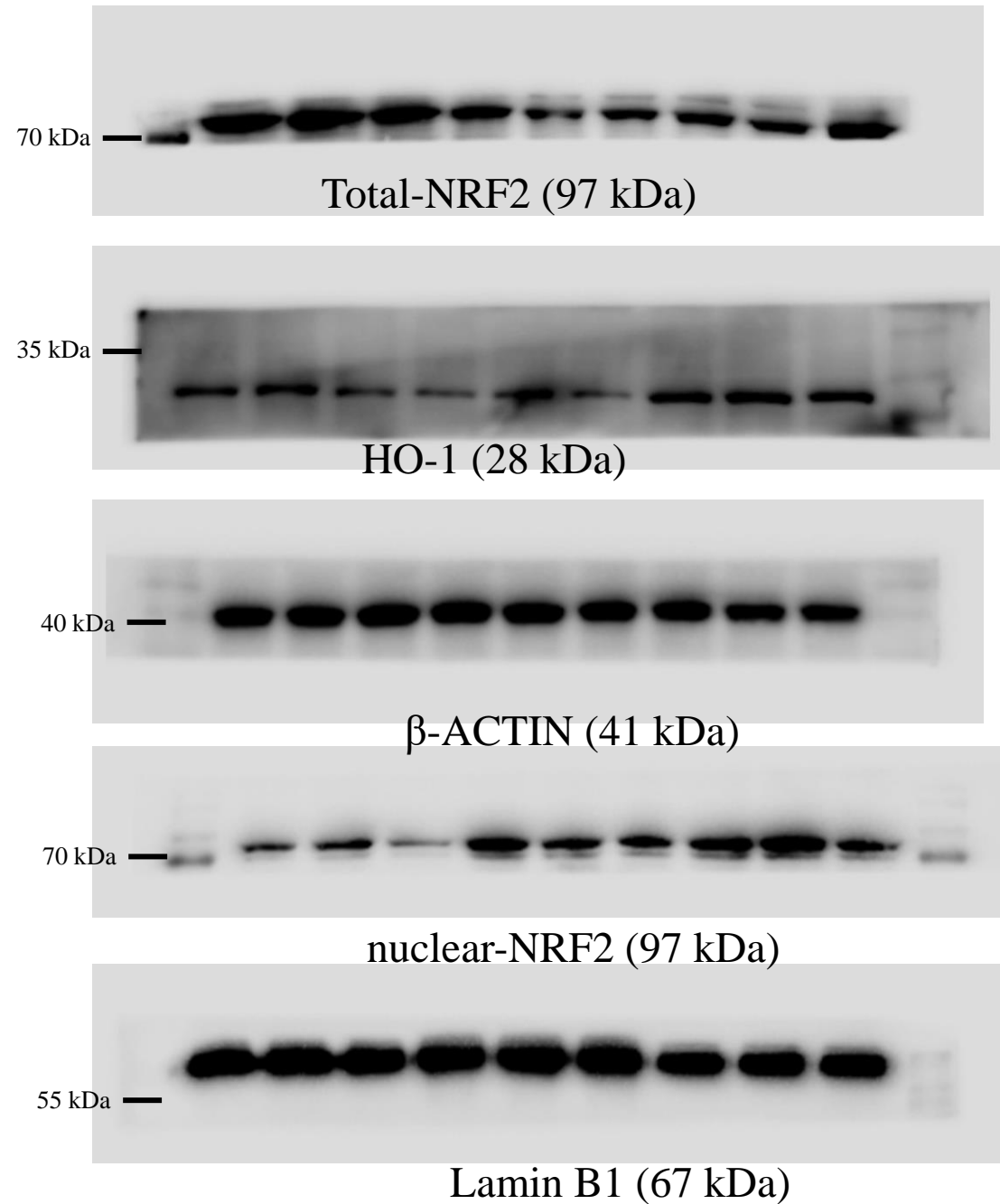

Figure 5B

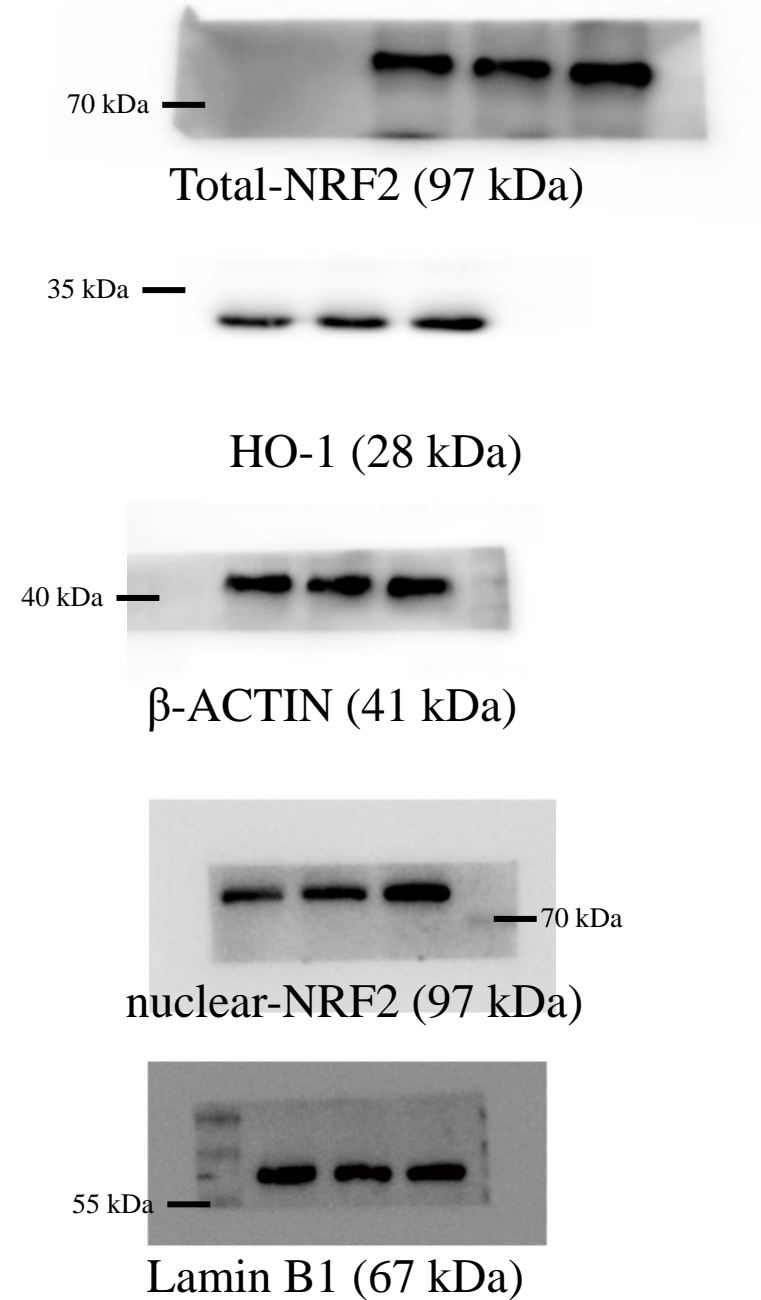

Figure 6F

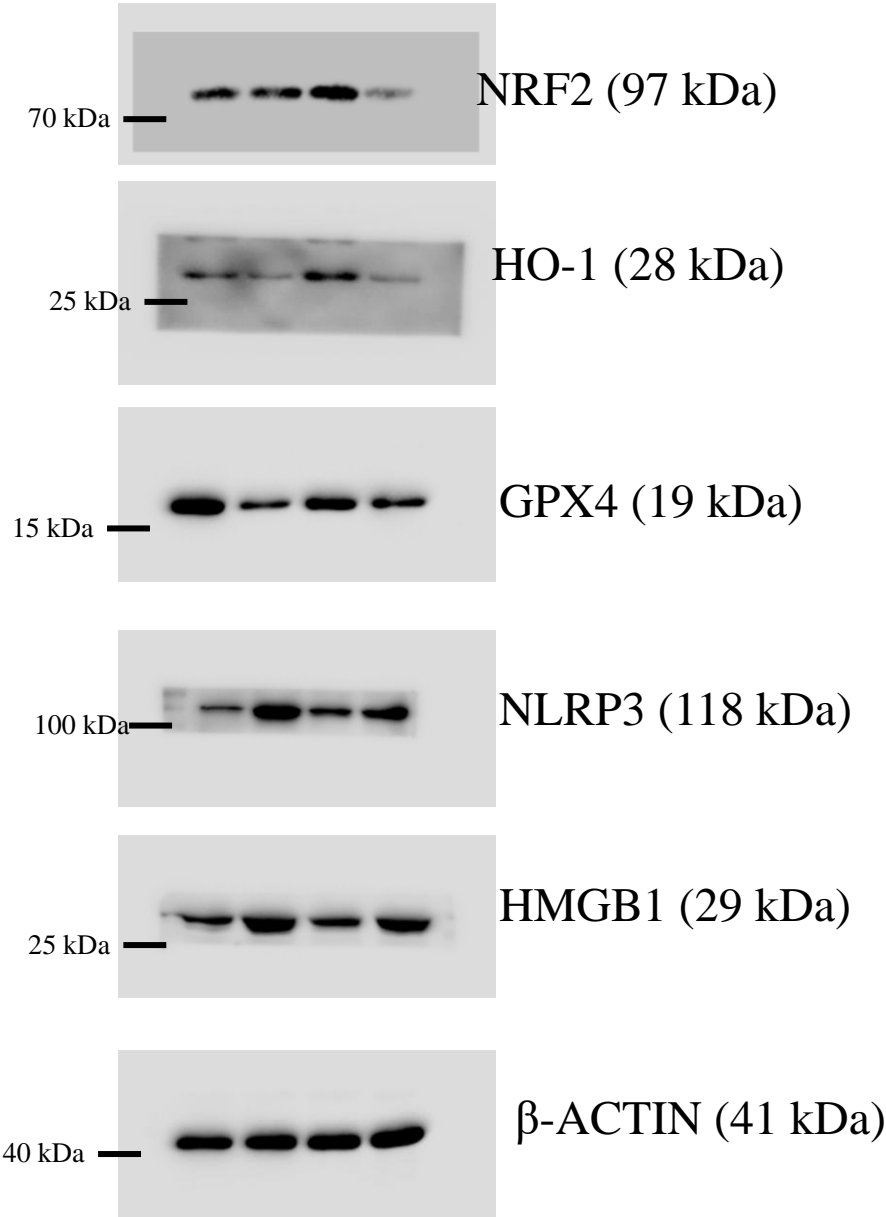

Figure 6G

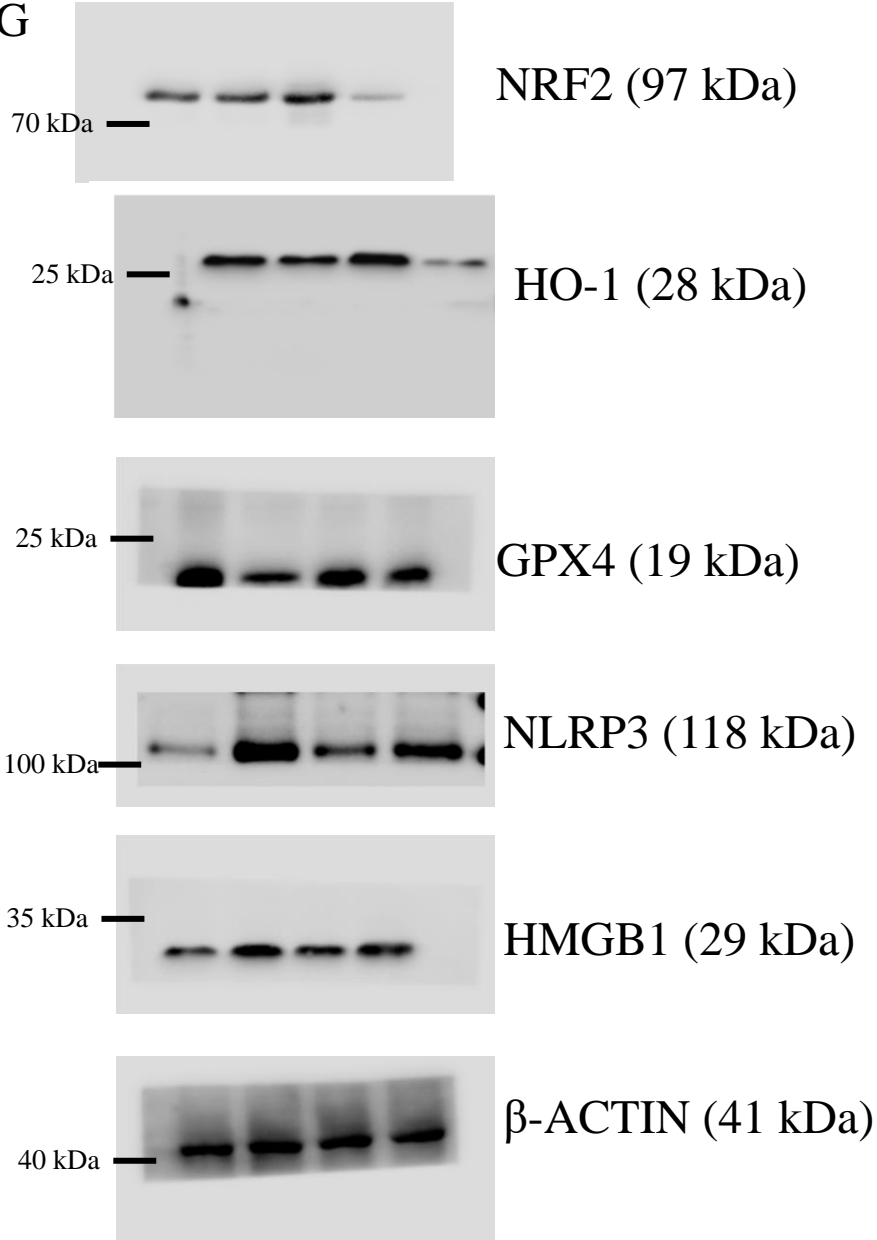

Supplement: Supplementary file 2 — Data S2. [file CNS-30-e14456-s002.pdf]
